# Otogenic Lateral Sinus Thrombosis: Controversies and Current Management Strategies

**DOI:** 10.3390/medicina62061093

**Published:** 2026-06-04

**Authors:** Alexandra Madalina Bizdu-Branovici, Luana Gherasie, Maria Denisa Zica, Andreea Rusescu, Irina Gabriela Ionița, Razvan Hainaroșie, Viorel Zainea

**Affiliations:** 1 General Medicine, “Carol Davila” University of Medicine and Pharmacy, 8th Eroii Sanitari Boulevard, 050474 Bucharest, Romania; alexandra.bizdu-branovici@drd.umfcd.ro (A.M.B.-B.); denisa.zica@drd.umfcd.ro (M.D.Z.); irina.ionita@umfcd.ro (I.G.I.); razvan.hainarosie@umfcd.ro (R.H.); viorel.zainea@umfcd.ro (V.Z.); 2 “Prof. Dr. D. Hociotă” Institute of Phonoaudiology and Functional ENT Surgery, 21st Mihail Cioranu Street, 050751 Bucharest, Romania

**Keywords:** otogenic lateral sinus thrombosis, mastoiditis, anticoagulation, cerebral venous sinus thrombosis, mastoidectomy, pediatric otology

## Abstract

*Background*: Otogenic lateral sinus thrombosis (OLST) is a rare but potentially life-threatening intracranial complication of middle-ear infection. Despite advances in imaging, antimicrobial therapy and otologic surgery, optimal management—particularly anticoagulation—remains controversial, and no standardized clinical guideline is available. *Methods*: A structured narrative review was conducted using PubMed for English-language human studies published between 1 January 2015 and 31 January 2025. The search was repeated and documented during revision on 12 May 2026. Four searches were run separately; retrieved records were manually merged, and duplicate record occurrences were removed using PMID. The searches retrieved 83 records before deduplication; after removal of 19 duplicates, 64 unique records remained for title and abstract screening. Single case reports and review articles were excluded from the primary descriptive synthesis. SANRA principles guided review quality and transparency. Seven eligible studies comprising 140 confirmed OLST patients were analyzed descriptively; selected clinically relevant but non-comparable publications were retained for contextual discussion. *Results*: Most included cohorts were pediatric; one study included both pediatric and adult patients. Clinical presentation was heterogeneous and often attenuated by prior antibiotic exposure. Contrast-enhanced CT was frequently used initially, whereas MRI/MRV was most informative for confirming thrombus extent and follow-up. Broad-spectrum intravenous antibiotics and surgical source control represented core treatment. Anticoagulation was reported in six studies, most often with low molecular weight heparin, but indications and duration varied substantially. Outcomes were generally favorable, although visual impairment, hearing loss, behavioral sequelae and incomplete radiological recanalization were reported. *Conclusions*: OLST management should be individualized according to disease severity, thrombus extent, septic status, and patient-specific risk factors. Antibiotics and source control are essential, while anticoagulation should be considered selectively. A practical management algorithm is proposed, but prospective multicenter data are needed.

## 1. Introduction

Otogenic lateral sinus thrombosis (OLST) represents a rare but severe intracranial complication of acute or chronic otitis media and mastoiditis. In the otologic literature, the term lateral sinus thrombosis is frequently used as an umbrella term that includes sigmoid sinus thrombosis and, in some cases, extension to the transverse sinus, jugular bulb or internal jugular vein. In this review, OLST is retained as the general term, while individual sinus involvement is reported according to the terminology used in each included study [1,2].

The pathogenesis of OLST is primarily related to the anatomical proximity between the mastoid air cell system and the sigmoid sinus. Acute coalescent mastoiditis, chronic otomastoiditis, cholesteatoma or persistent middle-ear infection may lead to perisinus inflammation, bony erosion, endothelial injury, platelet aggregation and septic thrombus formation. Thrombus propagation can extend to the transverse sinus and internal jugular vein and may contribute to intracranial hypertension, papilledema, abducens nerve palsy, venous infarction, hydrocephalus or septic embolic complications [1,2,3].

The microbiological background differs according to the underlying otologic disease. Acute otitis media and acute mastoiditis are commonly associated with *Streptococcus pneumoniae*, *Streptococcus pyogenes*, *Haemophilus influenzae*, *Staphylococcus aureus* and anaerobic pathogens, whereas chronic suppurative otitis media and cholesteatomatous disease may involve *Pseudomonas aeruginosa*, mixed aerobic–anaerobic flora, and resistant organisms [3,4]. *Fusobacterium necrophorum* is particularly relevant in acute mastoiditis complicated by septic cerebral venous sinus thrombosis and may be associated with prolonged hospitalization, antibiotic exposure, and recovery [5].

Although antibiotic use has reduced the incidence and mortality of otogenic intracranial complications, it has also modified the classical clinical picture. High spiking fever and overt septicemia are now less consistently observed, and many patients present after partial outpatient antibiotic treatment with attenuated systemic symptoms. Persistent headache, otalgia or otorrhea, diplopia, papilledema, lethargy, vomiting, nuchal rigidity or failure to improve under appropriate treatment should prompt urgent evaluation for intracranial extension [2,3,5].

The optimal management strategy remains controversial. Broad-spectrum intravenous antibiotics and surgical eradication of the otogenic focus are widely accepted, but the extent of mastoid surgery, the role of direct sinus exploration, the need for anticoagulation, the duration of anticoagulant therapy and the significance of radiological recanalization remain debated [2,3,5,6]. The aim of this structured narrative review is to synthesize contemporary evidence on diagnostic and therapeutic strategies for OLST and to propose a practical management framework for daily clinical decision-making.

## 2. Materials and Methods

This article was designed as a structured narrative review. The purpose was not to claim systematic completeness or to perform a meta-analysis, but to provide a transparent, clinically oriented synthesis of recent evidence regarding otogenic lateral/sigmoid sinus thrombosis. The manuscript was revised to avoid wording suggestive of a formal systematic review where such methodology was not applied.

The review was prepared in accordance with the Scale for the Assessment of Narrative Review Articles (SANRA), which was used as a methodological quality framework for the current narrative review. SANRA was not used as a risk of bias instrument for individual observational studies because the tool was developed to assess narrative review quality rather than primary study validity [7]. A completed SANRA self-checklist for the current review is provided as Supplementary Table S1.

A PubMed search was performed for English-language human studies published between 1 January 2015 and 31 January 2025. Because the exact day of the original search was not prospectively recorded during manuscript preparation, the search strategy was repeated and documented during revision on 12 May 2026. Four PubMed searches were run separately using the following free text PubMed queries: (1) cerebral venous sinus thrombosis mastoiditis; (2) lateral sinus thrombosis chronic otitis; (3) lateral sinus thrombosis mastoiditis; and (4) sigmoid sinus thrombosis otitis. These terms were chosen to capture both traditional otologic terminology and broader cerebral venous sinus thrombosis terminology used in pediatric, otolaryngological, radiological, and neurological publications.

**Figure 1:**
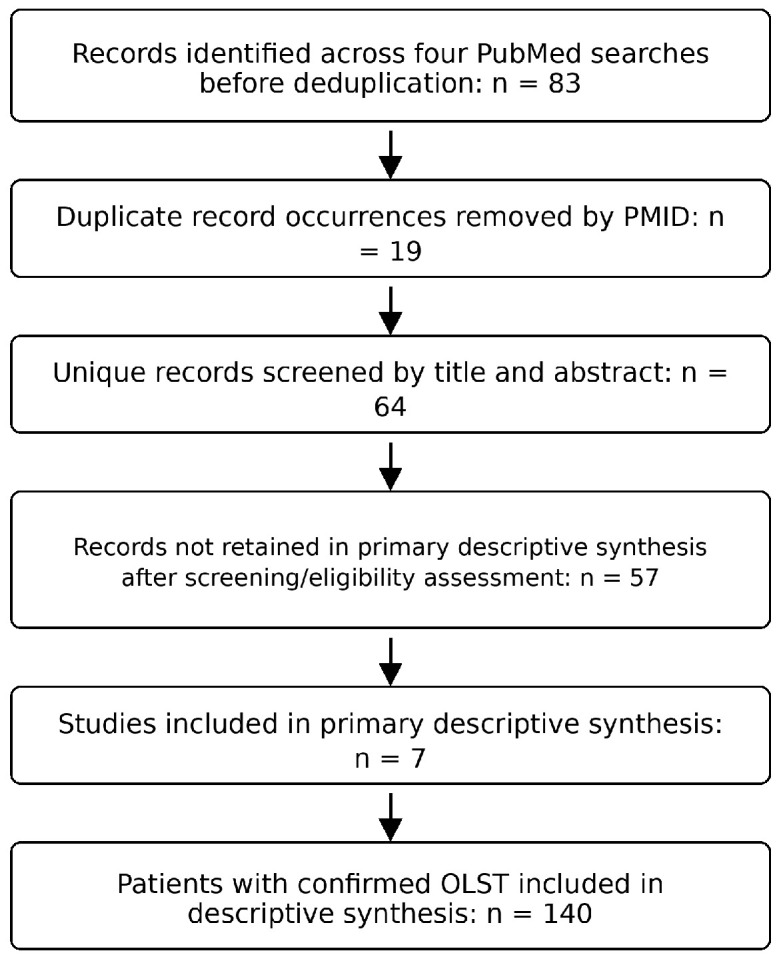
PRISMA-style study selection flow diagram for the structured narrative review. The figure summarizes PubMed search retrieval, manual deduplication by PMID, title/abstract screening, eligibility assessment, and final inclusion for the primary descriptive synthesis. OLST, otogenic lateral sinus thrombosis; PMID, PubMed identifier.

The records retrieved from the four searches were manually merged, and duplicate record occurrences were removed using PMID as the primary identifier. When necessary, title, author list and publication year were also checked to confirm duplicate status. The four searches retrieved 83 records before deduplication. After removal of 19 duplicate record occurrences, 64 unique PubMed records remained for title and abstract screening. All 64 unique records were screened by title and abstract for relevance to otogenic lateral/sigmoid sinus thrombosis. Records clearly unrelated to otogenic infection, lateral/sigmoid sinus thrombosis, mastoiditis or cerebral venous sinus thrombosis were excluded at the title/abstract level. Full-text assessment was performed for records considered potentially eligible for the primary descriptive synthesis or for contextual discussion. After applying the eligibility criteria, seven studies were included in the final primary descriptive synthesis, comprising 140 confirmed OLST patients. The study selection process is presented in Figure 1.

Studies were eligible if they reported pediatric or adult patients diagnosed with OLST secondary to acute or chronic otologic infection, including acute mastoiditis, chronic otitis media or cholesteatomatous otomastoiditis, either as an isolated complication or in association with other otogenic intracranial complications, provided that OLST-specific clinical, imaging, management or outcome data were extractable. Eligible domains included clinical presentation, imaging findings, sinus involvement, microbiology, antibiotic therapy, surgical management, anticoagulation, recanalization or clinical outcomes.

Single case reports were excluded to reduce anecdotal bias. Review articles were excluded from the primary synthesis to avoid duplication of cases, but selected reviews and guidelines were used for contextual discussion. Studies focused on non-otogenic cerebral venous sinus thrombosis, malignant otitis externa, external auditory canal cholesteatoma or unrelated intracranial complications were excluded. Studies were not excluded solely because some variables were not reported; instead, missing values were recorded as NR (not reported) to preserve transparency and avoid selective exclusion of the limited adult or mixed population evidence.

Study selection and data extraction were performed independently by two authors, with discrepancies resolved by consensus. Extracted variables included study year and country, number of OLST patients, age group, sex when available, otologic presentation, neurological findings, sinus involvement, microbiological data, antibiotic therapy, surgical procedures, anticoagulation regimen and duration, clinical outcomes, radiological recanalization and reported sequelae.

Due to heterogeneity in study design, patient population, outcome definitions, surgical indications, anticoagulation strategies, and imaging follow-up, meta-analysis was not appropriate. Results were therefore synthesized descriptively, with emphasis on clinically relevant patterns and areas of controversy. Patient counts and event counts were interpreted cautiously because several studies reported overlapping treatment categories or incomplete denominators.

## 3. Results

### 3.1. Study Selection and Evidence Base

Seven studies published between 2015 and 2021 met the eligibility criteria for the primary descriptive synthesis [5,8,9,10,11,12,13]. Together, they included 140 patients with OLST secondary to otologic infection. The corrected total of 140 patients was calculated by including only patients with confirmed otogenic lateral/sigmoid sinus thrombosis. In Vergadi et al., only the five patients with cerebral venous sinus thrombosis were included in the primary descriptive dataset; the remaining patients from the broader acute mastoiditis cohort without OLST were excluded. Additional clinically relevant publications identified during screening were reviewed, but were not included in the primary descriptive dataset when they did not meet the predefined comparability requirements for the tabulated anticoagulation, follow-up imaging and recanalization analysis. These publications were retained as contextual evidence where appropriate.

Most included studies were pediatric, while Huang et al. provided rare mixed adult and pediatric data [13]. Huang et al. were retained for demographic and anatomical comparison, particularly because adult-specific evidence is scarce. However, because treatment, antibiotic, anticoagulation and outcome variables were incompletely reported, this study was not used for treatment-specific or outcome-specific conclusions; unavailable variables were recorded as NR.

The clinical characteristics, venous involvement and surgical procedures reported in the included studies are summarized in Table 1.

### 3.2. Clinical Presentation and Diagnosis

The widespread use of antibiotics has altered the classical presentation of OLST [3,6]. Many patients receive empirical treatment before hospital admission, leading to attenuated systemic symptoms and delayed recognition of complications. Neurological manifestations such as abducens nerve palsy, papilledema or signs of intracranial hypertension remain important diagnostic indicators, but are inconsistently present. Therefore, persistent symptoms despite adequate therapy should raise suspicion for intracranial complications [5,8,9,10,11,12,13].

Diagnosis of OLST relies on a combination of clinical findings and imaging techniques [2,3,8,9,10,11,12,13,14,15]. Contrast-enhanced CT is frequently used as an initial modality, particularly in emergency settings, because it can identify mastoid opacification, coalescent mastoiditis, bony erosion, subperiosteal abscess, and associated otogenic or intracranial complications [2,3,4,8,9,10,11,12]. MRI combined with MR venography is currently considered the most informative modality for confirming venous sinus thrombosis, determining thrombus extension, and identifying associated intracranial complications [2,3,8,9,10,11,12,13,14,16]. Follow-up imaging is commonly performed to assess thrombus evolution and recanalization, although the relationship between radiological recanalization and clinical outcome remains debated [2,8,9,10,11,12,13,14].

### 3.3. Microbiology and Antibiotic Therapy

Microbiological reporting was incomplete. When available, isolated organisms included *Proteus mirabilis*, *Pseudomonas aeruginosa*, *group A Streptococcus pyogenes*, *Streptococcus* spp., *Haemophilus influenzae*, *Streptococcus mitis*, *Streptococcus pneumoniae*, anaerobic Gram-negative coccobacilli and *Fusobacterium necrophorum* [5,10,11,12]. Coudert et al. emphasized the relevance of *Fusobacterium necrophorum* in acute mastoiditis associated with sinus thrombosis and its association with prolonged treatment and recovery [5].

All studies that reported treatment used broad-spectrum intravenous antibiotics. Third-generation cephalosporins were frequently combined with anaerobic coverage, including metronidazole or clindamycin. Vancomycin, teicoplanin, ceftazidime, meropenem or other agents were used in selected cases according to clinical severity, local practice or microbiological results [5,8,9,10,11,12].

### 3.4. Surgical Management

Surgical management was reported in a heterogeneous manner. Across the included studies, procedures included myringotomy with or without tympanostomy tube placement, mastoidectomy, mastoidectomy with sinus exposure, perisinus empyema drainage, craniotomy for abscess or epidural collection, canal-wall-down mastoidectomy in selected cholesteatomatous or advanced disease and lumbo-peritoneal shunting for intracranial hypertension (Table 1) [5,8,9,10,11,12,13].

The contemporary trend favors surgical eradication of the otogenic source while avoiding routine thrombectomy or internal jugular vein ligation. Direct sinus exploration was reserved for selected cases, such as perisinus abscess, persistent septicemia, progressive thrombus extension, or failure of clinical improvement despite appropriate medical and otologic management.

### 3.5. Anticoagulation, Recanalization, and Outcomes

Anticoagulation regimens, antibiotic therapy and reported clinical and radiological outcomes are summarized in Table 2.

Anticoagulation was reported in six of the seven included studies [5,8,9,10,11,12]. Low molecular weight heparin was the most frequently reported agent. Unfractionated heparin, warfarin and acenocoumarol were used in selected cases or as continuation therapy. Anticoagulant classes were not combined as mutually exclusive patient counts because several studies reported transitions or combined regimens.

Reported anticoagulation duration ranged from approximately 8 to 48 weeks among studies providing extractable duration data (Table 2). Because duration was inconsistently reported and anticoagulant regimens sometimes involved transitions between agents, no pooled mean duration was calculated. Some authors recommended fixed durations, while others continued treatment until radiological recanalization or clinical stabilization. However, incomplete recanalization was reported even after prolonged anticoagulation and favorable clinical recovery was also described in patients without complete radiological resolution [5,8,9,10,11,12].

Among studies reporting clinical outcomes, recovery was generally favorable. The initial dataset reported 106 patients with full recovery/no sequelae in studies with extractable outcome data, while reported sequelae included impaired visual acuity, optic nerve atrophy, behavioral disorders or hyperactivity, and severe sensorineural hearing loss. Huang et al. did not provide sufficient outcome data for inclusion in this outcome count [13].

## 4. Discussion

This structured narrative review shows that contemporary OLST evidence remains dominated by small retrospective pediatric cohorts, heterogeneous reporting and limited adult data. The available literature supports a consistent therapeutic foundation: early recognition, urgent imaging, broad-spectrum intravenous antibiotics and surgical control of the otogenic source. In contrast, anticoagulation, duration of therapy and interpretation of recanalization remain incompletely standardized.

The clinical diagnosis remains challenging in the antibiotic era. Partial treatment before hospitalization may reduce fever and systemic toxicity, while neurological or ophthalmological signs may be intermittent or absent early [2,3,5,6]. Therefore, persistent otologic symptoms associated with headache, diplopia, papilledema, lethargy, vomiting, neck stiffness or visual complaints should lower the threshold for MRI/MRV after initial CT evaluation [2,3,5,8,9,10,11,12,13,14,16].

Antibiotic therapy should be immediate and broad enough to cover the expected spectrum of acute mastoiditis and chronic otologic infection. In acute otitis media and acute mastoiditis, common pathogens include *Streptococcus pneumoniae*, *Streptococcus pyogenes*, *Haemophilus influenzae*, *Staphylococcus aureus* and anaerobes. In chronic suppurative otitis media or cholesteatoma, *Pseudomonas aeruginosa*, mixed aerobic–anaerobic flora and resistant organisms become more relevant [3,4].

The included studies most frequently reported third-generation cephalosporins combined with anaerobic coverage, with glycopeptides or antipseudomonal agents added according to disease severity, chronicity, local microbiology, or culture results. *Fusobacterium necrophorum* deserves particular attention because it has been associated with sinus thrombosis in acute mastoiditis and a more prolonged course [5].

Surgical source control remains fundamental. The goal of surgery is eradication of middle-ear and mastoid infection, drainage of purulent collections, ventilation of the middle ear when appropriate and prevention of further septic propagation. Mastoidectomy, with or without tympanostomy tube placement, was the most frequent intervention in the included studies, although some children with selected disease patterns were treated with tympanostomy alone or even without surgery [5,9].

A conservative philosophy toward the thrombosed sinus itself is increasingly reflected in contemporary practice [6,14,15,17,18,19,20,21]. Routine sinus incision, thrombectomy and internal jugular vein ligation are no longer standard. These procedures may be reserved for persistent septicemia, perisinus abscess, progressive thrombus despite adequate therapy or cases in which intraoperative findings demand direct management [6,10,14,15].

This interpretation is consistent with earlier systematic and institutional reviews that emphasized multidisciplinary management and a gradual move away from aggressive sinus surgery in selected clinically stable patients [14,15].

Anticoagulation is the central controversy in OLST. General cerebral venous thrombosis (CVT) guidance supports anticoagulation in many patients without contraindications, including selected cases with venous hemorrhagic lesions, because the therapeutic objective is to prevent thrombus propagation, promote recanalization and reduce venous pressure [16,22,23]. However, OLST is a distinct septic, otogenic condition in which surgical source control and antimicrobial therapy are essential, and evidence is derived primarily from retrospective pediatric case series rather than randomized trials.

For this reason, recommendations from general CVT should not be transferred mechanically to OLST, as general CVT guidance is derived from broader cerebral venous thrombosis populations, whereas OLST represents an otogenic, frequently septic, source-related condition [2,3,16,22,23]. In otogenic disease, the decision must also consider septic thrombus biology, mastoid source control, intracranial abscess or empyema, recent otologic or neurosurgical procedures, hemorrhagic risk and the presence of intracranial hypertension or thrombus propagation [5,8,9,10,11,12,14,24,25]. The most defensible conclusion is not that anticoagulation is unnecessary, but that its use should be individualized and justified by subgroup-specific risk [2,3,8,9,10,11,12,14,16,22,23,24,25].

Otogenic-specific reappraisals and recent post-search pediatric mastoiditis-associated CSVT data support the same cautious interpretation: anticoagulation is frequently used, but the decision remains dependent on thrombus extent, venous drainage anatomy, intracranial hypertension, postoperative bleeding risk, and multidisciplinary assessment rather than on a universal rule [24,25].

Foundational pediatric series and otologic reference sources were retained as the contextual literature because they document the historical evolution of OLST management, recanalization follow-up, thrombophilia evaluation and the progressive shift away from routine sinus thrombectomy or internal jugular vein ligation [17,18,19,20,26,27,28]. Additional clinically relevant, but non-included series further illustrate the heterogeneity of practice. Ryan et al. [21] described pediatric OLST management with antibiotics, otologic surgery, and selective anticoagulation, whereas Raja et al. [15] reported a more surgically oriented mixed-age institutional approach, including internal jugular vein ligation in selected patients and no anticoagulation exposure. These reports were retained for contextual discussion only and did not contribute patients to the 140-patient primary descriptive dataset because their reporting structure and management paradigms did not allow standardized comparison with the predefined extraction framework for anticoagulation strategy, treatment duration, follow-up imaging and recanalization outcomes.

The recent systematic review by Picton et al. provides important post-search contextual evidence and should be interpreted alongside, rather than pooled with, the present 140-patient primary descriptive dataset [29]. Picton et al. searched Medline, Embase, Emcare, Cochrane and ClinicalTrials.gov from inception to November 2024 and included 68 papers, comprising 43 case reports and 25 case series with 149 patients. They reported surgery in 88.5% of patients, anticoagulation in 54.8%, complete recanalization in 65% of reimaged patients, and long-term complications in 9.4%. Their conclusion that antibiotics, surgery and anticoagulation were associated with favorable outcomes is broadly concordant with the therapeutic principles identified in the present review. However, because their synthesis included case reports and non-comparative case series, it does not eliminate the need for individualized anticoagulation decisions. Importantly, Picton et al. found no additional benefit from incision and drainage of the sigmoid sinus and reported a higher complication signal with this approach, supporting the contemporary preference for otologic source control while avoiding routine direct sinus intervention.

Radiological recanalization is an important follow-up parameter, but current evidence does not establish it as the only meaningful endpoint [2,8,9,10,11,12,13,14,17,18,19]. Some patients improve clinically despite incomplete or absent recanalization, while neurological, visual or auditory sequelae may occur despite radiological resolution [8,9,10,11,12,17,18,19]. Clinical recovery, neurological examination, ophthalmological status, hearing outcome, inflammatory response and control of the otogenic source should therefore be interpreted together with imaging [2,8,9,10,11,12,13,14,17,18,19].

Follow-up MRI/MRV is commonly performed at approximately 3–6 months when reported, but protocols vary across studies [2,8,9,10,11,12,13,14,17,18,19]. Earlier repeat imaging is justified if symptoms worsen, intracranial pressure persists, thrombus propagation is suspected, or anticoagulation decisions depend on radiological evolution [8,9,10,11,12,16,17,18,19].

## 5. Proposed Management Algorithm

**Figure 2:**
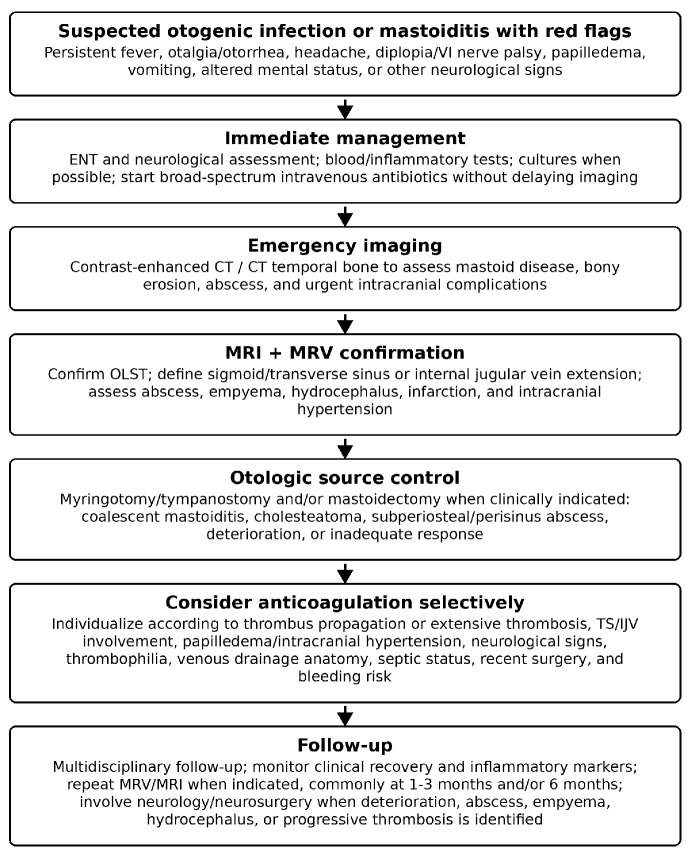
Proposed practical management algorithm for otogenic lateral sinus thrombosis. The algorithm synthesizes available evidence from the included studies and current principles of cerebral venous thrombosis management. It is intended as a practical clinical framework and not as a formal guideline. Treatment decisions should be individualized according to disease severity, thrombus extension, septic status, intracranial complications, bleeding risk, thrombophilia and multidisciplinary assessment. CT, computed tomography; IJV, internal jugular vein; MRI, magnetic resonance imaging; MRV, magnetic resonance venography; OLST, otogenic lateral sinus thrombosis; TS, transverse sinus.

Based on the revised synthesis, OLST management should proceed through a staged multidisciplinary pathway: early suspicion, emergency imaging, MRV confirmation, immediate antibiotics, surgical source control, individualized anticoagulation assessment, and structured clinical and radiological follow-up (Figure 2) [2,3,5,8,9,10,11,12,13,14,15,16,17,18,19,24,25].

## 6. Future Directions

The rarity of OLST makes randomized trials unlikely in the near future, but multicenter prospective registries are feasible and urgently needed. Future studies should use standardized definitions for sinus involvement, septic versus non-septic thrombosis, intracranial hypertension, recanalization, clinical recovery, visual outcomes, hearing outcomes and anticoagulation-related complications.

A minimal OLST registry should capture age, otologic disease type, microbiology, CT and MRI/MRV findings, surgical procedure, anticoagulant agent, timing, dose, duration, bleeding complications, recanalization timing and functional outcomes. Such datasets would allow clinically meaningful subgroup analyses and could help define when anticoagulation is beneficial rather than simply documenting that practice varies.

## 7. Limitations

This review has several limitations. First, the primary search was restricted to PubMed and English-language human studies, which may have excluded relevant reports indexed elsewhere. Second, although the PubMed search was repeated and documented during revision, this article remains a structured narrative review rather than a formal systematic review or meta-analysis; therefore, the findings should be interpreted as a clinically oriented synthesis rather than a pooled estimate of effect. Third, the included evidence consists predominantly of retrospective pediatric cohorts with small sample sizes; adult evidence is scarce and largely dependent on one mixed population study retained mainly for demographic and anatomical comparison. Fourth, publication bias is likely because complicated or successfully managed cases may be preferentially reported.

Fifth, outcome reporting was heterogeneous, with inconsistent denominators for sinus involvement, surgery, anticoagulant exposure, imaging follow-up and recanalization. Sixth, anticoagulation strategies were variable and often reported as treatment exposures rather than mutually exclusive patient-level groups, limiting quantitative comparison. Seventh, some included studies provided incomplete treatment or outcome data; these variables were recorded as NR and were not used to support domain-specific conclusions. Eighth, the primary dataset included studies published between 2015 and 2021, despite the updated PubMed search covering 2015 to 2025. The absence of additional eligible primary cohort studies after 2021 reflects the rarity of OLST, the exclusion of single case reports and review articles from the primary dataset and the limited availability of standardized cohort-level data. Recent contextual publications and guidelines were discussed where relevant, but were not included in the 140-patient primary descriptive dataset.

## 8. Conclusions

Otogenic lateral sinus thrombosis remains a rare but clinically important complication of acute or chronic otologic infection. The evidence supports urgent recognition, multimodal imaging, broad-spectrum intravenous antibiotics and surgical eradication of the otogenic source as the core of management.

The role of anticoagulation remains unresolved. Current data do not support indiscriminate routine anticoagulation for all patients, but they do support selective use in clinically meaningful subgroups, including thrombus propagation, extensive transverse sinus or internal jugular vein involvement, intracranial hypertension, papilledema, neurological signs, thrombophilia or compromised venous drainage. Conversely, stable isolated sigmoid sinus thrombosis after effective source control may be managed individually with close follow-up when bleeding risk is high.

Standardized multicenter data are required to establish evidence-based protocols for diagnosis, surgery, anticoagulation indication and duration, recanalization assessment, and long-term neurological, visual and auditory outcomes.

## Figures and Tables

**Table 1:** Included studies and key clinical characteristics of patients with otogenic lateral sinus thrombosis.

Study	N	Age	M/F	Otologic Symptoms	Neurological Findings	Sinus Involvement	Surgical Treatment
Zanoletti et al. [8]	8	2–7 y	4/4	Ear pain, fever	Diplopia, photophobia, lethargy, papilledema, headache	8 SS; 6 TS	Mastoidectomy (3); sinus opening (1); myringocentesis (2); transtympanic drainage (2)
Scorpecci et al. [9]	25	1–16 y	17/8	Ear pain, acute mastoiditis, otorrhea	Diplopia, papilledema, headache, nuchal rigidity	SS (17); SS+TS (4); SS+cavernous sinus (1); SS+sagittal sinus (1); SS+jugular bulb (2)	Mastoidectomy + tympanostomy tube (16); craniotomy/abscess evacuation (3)
Coutinho et al. [10]	16	2–16 y	7/9	Ear pain, fever, otorrhea	Headache, lethargy, nausea/vomiting, diplopia, seizures, papilledema, neck stiffness	SS (4); TS (2); SS+TS (6); SS+TS+IJV (4)	Mastoidectomy ± tube (16); perisinus empyema drainage (3); sinus exposure (7); craniotomy (5)
Coudert et al. [5]	52	2–18 y	NR	NR	NR	SS (26); SS+IJV (26)	Mastoidectomy (26); tympanostomy tube (13); no surgery (13)
Trapani et al. [11]	7	0–16 y	5/2	Ear pain, otorrhea, fever	Headache, diplopia, dizziness, papilledema, lethargy, nausea/vomiting	SS (1); SS+TS (3); TS+IJV (1); SS+TS+IJV (2)	Mastoidectomy (3); lumbo-peritoneal shunt (2); canal-wall-down mastoidectomy + tympanoplasty (1)
Vergadi et al. [12]	5 (cohort of 20 children with acute mastoiditis)	0–16 y	NR for OLST subset	Fever	Lethargy, diplopia, headache, ataxia, papilledema	SS (2); SS+TS (3); SS+IJV (1)	Myringotomy (1); mastoidectomy (3); no surgery (1)
Huang et al. [13]	27	Mixed pediatric/adult	NR	Ear pain, fever, otorrhea	Facial palsy, vertigo, headache	SS (9); SS+TS (9); SS+IJV (9)	NR

Abbreviations: SS, sigmoid sinus; TS, transverse sinus; IJV, internal jugular vein; NR, not reported. Patient counts refer to confirmed OLST cases only. For Vergadi et al. [12], only the OLST/CVST subset (*n* = 5) was counted; sex distribution for this subset was not separately reported. Huang et al. [13] were retained for demographic and anatomical comparison but not for treatment-specific or outcome-specific conclusions where data were NR.

**Table 2:** Anticoagulation regimens, antibiotic therapy and clinical/radiological outcomes in patients with otogenic lateral sinus thrombosis.

Study	Anticoagulation	Duration	Antibiotics	Duration	Clinical Outcomes	Radiological Outcomes
Zanoletti et al. [8]	LMWH	12 weeks	Ceftriaxone + metronidazole	10 days	8 full recovery	6 complete; 1 partial; 1 none
Scorpecci et al. [9]	LMWH	8 weeks	Ceftriaxone	NR	24 recovery; 1 visual deficit	20 complete; 3 no follow-up; 2 NR
Coutinho et al. [10]	UFH/LMWH/warfarin	48 weeks	Ceftriaxone ± metronidazole/clindamycin; ceftazidime + vancomycin in selected cases	24 days	12 recovery; neuro-ophthalmological/behavioral sequelae (4)	7 complete; 3 partial; 6 no follow-up
Coudert et al. [5]	LMWH/UFH	NR	Cephalosporin + anaerobic coverage	~31 days	52 recovery	32 complete; 13 partial; 5 no follow-up
Trapani et al. [11]	LMWH/oral anticoagulants	24 weeks	Cephalosporin + glycopeptide	NR	5 recovery; 2 sequelae	7 complete
Vergadi et al. [12]	LMWH	12 weeks	Ceftriaxone + vancomycin + metronidazole	NR	5 recovery	3 complete; 2 NR
Huang et al. [13]	NR	NR	NR	NR	NR	NR

Abbreviations: LMWH, low molecular weight heparin; UFH, unfractionated heparin; NR, not reported. Treatment categories are not mutually exclusive when studies reported transitions or combined regimens. For Vergadi et al. [12], only the OLST/CVST subset was counted. Huang et al. [13] were not used for treatment-specific or outcome-specific conclusions because the relevant data were NR.

## Data Availability

All data supporting the findings of this review are available within the article and its tables.

## Supplementary Materials

The following supporting information can be downloaded at: https://www.mdpi.com/article/10.3390/medicina62061093/s1, Table S1. SANRA-guided self-checklist for the current narrative review; Table S2. Clinically relevant publications were assessed but not included in the primary descriptive dataset.
