# Supplementary material for: Otogenic Lateral Sinus Thrombosis: Controversies and Current Management Strategies
Source: Medicina (Kaunas). 2026 Jun 4;62(6):1093. doi: 10.3390/medicina62061093 (PMC13302785; doi:10.3390/medicina62061093)
Supplement: Supplementary file 1 [file medicina-62-01093-s001.zip › medicina-4298132-supplementary.pdf]

Supplementary Materials

Otogenic Lateral Sinus Thrombosis: Controversies and Current Management Strategies

Table S1. SANRA-guided self-checklist for the current narrative review

SANRA item	Criterion	Score (0-2)	How addressed in the review
1	Justification of the article's importance for the readership	2	The review addresses a rare but severe otogenic intracranial complication relevant to otolaryngology, pediatrics, neurology, radiology, and infectious diseases.
2	Statement of concrete aims or formulation of questions	2	The Introduction states the aim of synthesizing diagnostic and therapeutic strategies for OLST and proposing a practical management framework.
3	Description of the literature search	2	The Methods report database, publication interval, search date, exact search strings, deduplication method, screening, eligibility criteria, and final study count.
4	Referencing	2	Statements are supported by primary cohort studies, contextual reviews, otologic references, and current CVT guidance; primary and contextual evidence are separated.
5	Scientific reasoning	2	The Discussion differentiates general CVT guidance from

			otogenic, septic, source-related OLST and emphasizes evidence limitations.
6	Appropriate presentation of data	2	Tables summarize clinical characteristics, treatment, anticoagulation, and outcomes, while missing variables are marked as NR and not used for domain-specific conclusions.

Total SANRA self-check score: 12/12. SANRA was used to assess the quality and transparency of the narrative review, not as a risk-of-bias instrument for individual observational studies.

Table S2. Clinically relevant publications assessed but not included in the 140-patient primary descriptive dataset

Publication	Publication type/focus	Reason for not including in the primary descriptive dataset	Use in manuscript
Ryan et al., 2016 (PMID: 26991219)	Retrospective pediatric case series	Clinically relevant but not sufficiently comparable with the predefined extraction framework for anticoagulation duration, standardized imaging follow-up, and recanalization outcomes.	Contextual discussion of pediatric management and historical/institutional practice.
Raja et al., 2018 (PMID: 29983756)	Retrospective mixed-age case series	Clinically heterogeneous cohort with institution-specific surgical management, including internal jugular vein ligation in selected cases; no anticoagulation exposure and incomplete standardized imaging follow-up/recanalization data.	Contextual discussion of surgical source control and changing management trends.

Fiordelisi et al., 2025 (PMID: 39820761)	Retrospective pediatric acute mastoiditis cohort	Focused on clinical characteristics and predictive factors for thrombotic complications in acute mastoiditis rather than providing OLST-specific treatment, anticoagulation duration, surgical treatment, follow-up imaging, and recanalization variables comparable with the primary dataset.	Contextual recent evidence on predictors of thrombotic complications.
Sutter et al., 2023 (PMID: 37778041)	Pediatric septic CVST in sinogenic/otogenic intracranial infections	Mixed sinogenic and otogenic infection cohort without separately extractable OLST-specific patient-level data for the predefined otogenic treatment/recanalization framework.	Contextual evidence for anticoagulation in septic CVST.
Chen et al., 2025 (PMID: 39562726)	Otitic hydrocephalus/papilledema treatment study	Focused on intracranial pressure and visual outcomes rather than OLST-specific surgical, anticoagulation, and recanalization variables.	Contextual discussion of intracranial hypertension and follow-up.
Shiran et al., 2024 (PMID: 38724201)	Radiological/microbiological prediction study in complicated acute mastoiditis	Focused on CT venography prediction of Fusobacterium necrophorum as a causative agent rather than OLST-specific treatment and outcome comparison.	Contextual discussion of microbiology and imaging.
Lu et al., 2023 (PMID: 37060317)	Systematic review and meta-analysis	Review-level evidence without new original cohort data eligible for the primary descriptive dataset; excluded from patient counts to avoid duplication.	Contextual discussion only.

NR, not reported; OLST, otogenic lateral sinus thrombosis; CVST, cerebral venous sinus thrombosis. Publications listed here were assessed for relevance but did not contribute patients to the 140-patient primary descriptive dataset.
